# Impact of APOE4‐related dementia risk in underrepresented groups from the *All of Us* research program

**DOI:** 10.1002/alz.70245

**Published:** 2025-06-11

**Authors:** Valentina Ghisays, Ehsan Khajouei, Ignazio S. Piras, Michael H. Malek‐Ahmadi, Hillary D. Protas, Dhruman D. Goradia, Yinghua Chen, Marcus Naymik, Donald Saner, Olivia J. Veatch, Clayton O. Mansel, Yi Su, Matthew J. Huentelman, Jason H. Karnes, Eric M. Reiman

**Affiliations:** ^1^ Banner Alzheimer's Institute Phoenix Arizona USA; ^2^ Department of Pharmacy Practice and Science, R. Ken Coit College of Pharmacy University of Arizona Tucson Arizona USA; ^3^ Early Detection and Prevention Division Translational Genomics Research Institute Phoenix Arizona USA; ^4^ Department of Cell Biology and Physiology University of Kansas Medical Center Kansas City Kansas USA

**Keywords:** Alzheimer's disease, APOE genotype, electronic health records, social determinants of health underrepresented groups

## Abstract

**INTRODUCTION:**

Longitudinal electronic health records (EHRs), “dementia” diagnostic codes, and genetic data from *All of Us* were used to see if there is a higher risk of dementia and an attenuated impact of apolipoprotein *ε4* (APOE4) on Alzheimer's disease (AD) risk in Black and Hispanic/Latino groups.

**METHODS:**

Participants included 9,784 Hispanic/Latinos, 14,937 Non‐Hispanic Blacks (NHB), and 60,388 Non‐Hispanic Whites (NHWs) ≥age 60 without an initial dementia diagnosis.

**RESULTS:**

There was a significantly higher risk of developing a dementia diagnosis in Hispanic/Latino and NHB participants than NHW participants and comparably increased dementia hazard ratios in the Hispanic/Latino, NHB, and NHW APOE4 carriers than non‐carriers (hazard ratio [HR] [95% confidence interval {CI}] 1.54 [1.25–1.91], 1.25 [1.00–1.57], and 1.55 [1.40–1.72]).

**DISCUSSION:**

Compared to NHW individuals, Hispanic/Latino and NHB individuals are at higher risk of developing a “dementia diagnosis” and comparably higher risk in APOE4 carriers than non‐carriers. Plasma AD biomarkers could clarify the proportion of dementia cases with and without AD and the impact of APOE4 on AD in these groups.

**Highlights:**

- Hispanic/Latino and non‐Hispanic Black individuals have a higher risk of all‐cause dementia.
- The impact of apolipoprotein *ε4* (APOE4) on dementia risk was similar in the studied ethnoracial groups.
- Plasma biomarkers could inform the risk of Alzheimer's disease (AD) and impact of APOE4 in these groups.

## 1 BACKGROUND

Alzheimer's disease (AD) is the most common form of disabling cognitive impairment in older adults. There is an urgent need to understand, treat, and prevent this disorder. The understanding of genetic and non‐genetic risk factors has already begun to have a profound impact on this endeavor. The apolipoprotein *ε4* allele (APOE4) is the major susceptibility gene for developing idiopathic AD at older ages. The APOE gene has three common alleles (APOE *ε*2, *ε*3, and *ε*4), giving rise to six common genotypes (APOE 2/2, 2/3, 3/3, 2/4, 3/4, and 4/4).^1^, ^2^, ^3^, ^4^, ^5^, ^6^, ^7^, ^8^, ^9^, ^10^ In comparison to APOE 3/3, the most common APOE genotype, APOE4 is found in about 25% of the population and in about 60% of persons with the clinical and neuropathological diagnosis of AD, with each additional allele copy associated with a higher risk and earlier age at onset of AD biomarker changes, cognitive decline, and dementia.^1^, ^2^, ^3^, ^4^, ^5^, ^6^, ^7^, ^8^, ^9^, ^10^ Conversely, APOE *ε2* (APOE2) is the least common allele with each additional copy associated with a lower risk for AD.^6^, ^9^, ^11^ The influence of APOE4 on a person's AD risk, subsequent rate of clinical decline, and differential beneficial and adverse effects of the first AD disease‐modifying medications has begun to have a major impact on the scientific and clinical fight against the disease.^12^, ^13^

Importantly, race and ethnicity may also play a key role in determining effects of APOE with studies indicating potential differences in the association of APOE4 on AD endophenotypes and risk.^14^, ^15^, ^16^, ^17^, ^18^, ^19^, ^20^ Genetic associations of APOE with AD risk in different underrepresented groups (URGs) remains unclear with some studies raising the possibility that APOE4 has a smaller impact on the risk for AD in non‐Hispanic African American/Black (NHB) and Hispanic/Latino than in non‐Hispanic White (NHW) persons.^14^, ^15^, ^16^, ^17^, ^18^, ^19^, ^20^ This may be due to factors such as large heterogeneity in the Hispanic/Latino population, small sample sizes, other methodological limitations such as reliability of APOE genotyping data in meta‐analyses, or racial biases when assessing dementia.^21^, ^22^ Confirming that possibility in larger real‐world cohorts could have major implications for research and care in these URGs, as well as the effort to discover protective mechanisms that could be targeted by future AD‐modifying and prevention therapies.

We used electronic health record (EHR) data from the *All of Us* research program^23^, ^24^ to compare the relative risk of getting a dementia diagnosis in these URGs as well as the impact of APOE4 carriage and allelic dose on dementia hazard ratios.

## 2 METHODS

### 2.1 The *All of Us* research program

The *All of Us* Research Program is a national effort to build a diverse biomedical database to advance precision medicine. The program addresses the historical underrepresentation of certain populations in research, with over 77% of participants from underrepresented communities and 46% from racial and ethnic minority groups.^23^, ^24^ Participants enroll digitally or through a network of over 340 recruitment sites, including regional medical centers, federally qualified health centers, the Veterans Health Administration, and direct‐volunteer pathways. Informed consent is obtained through interactive digital modules, and participants contribute data through health surveys, physical measurements, biospecimens, and digital health devices.^25^ The dataset integrates EHRs from 34 contributing sites, capturing structured data such as billing codes, medication history, laboratory results, vital signs, and clinical encounters. Additional sources include personal and family medical histories, lifestyle surveys, and biospecimens (blood, urine, and saliva for DNA, RNA, and other biomarkers).^25^ Digital health data, including wearable device metrics, are also incorporated, with ongoing pilot studies exploring expanded data collection.^25^

### 2.2 APOE status determination

APOE genotypic data were extracted and tabulated for 245,366 participants in *All of Us* v7. We used short‐read whole genome sequencing (srWGS) data and defined APOE alleles by analyzing the single nucleotide polymorphisms (SNPs) rs429358 and rs7412.^26^, ^27^ The data now containing the six most common APOE genotypes including 2/2, 2/3, 3/3, 2/4, 3/4, and 4/4 were integrated into the cohort data leaving 206,150 unique person IDs with APOE genotype information.

### 2.3 Data preparation

We used unique identifier or person ID from each participant throughout the data preparation process, including querying the database, merging tables, and constructing the cohort. All International Classification of Disease (ICD‐9 and ICD‐10) codes related to a dementia diagnosis or associated drug prescriptions were used to identify cases of all‐type dementia and age‐matched controls. The first step involved creating the case dataset using the cohort builder tool embedded in the workbench. We extracted data separately from “drug exposure” and “condition occurrence” tables by running a general query for “dementia” as a keyword. This process captured data from all participants with EHRs who have been either diagnosed with any type of dementia (e.g., uncomplicated senile dementia, vascular dementia, AD, etc.) or prescribed “dementia‐related” medications (e.g., cholinesterase inhibitors and/or glutamate regulators such as donepezil, rivastigmine galantamine memantine, etc.). At the end of this step, information from both tables was merged and a new feature was created to track the earliest date on which either a dementia diagnosis or relevant medication prescription occurred. We ran a sensitivity analysis to assess the impact of excluding dementia‐related prescriptions without a corresponding dementia diagnostic code and postconcussion syndrome cases from our case definition. We note that prevalent cases of dementia were defined as the first timepoint in the EHR, and “mild cognitive disorder” or “amnestic mild cognitive disorder” were not part of the case definition used, only incident dementia diagnoses counted as events.

To construct the control dataset, we first queried the database with Structured Query Language (SQL) to identify and extract two key dates for each participant, their earliest and their most recent EHR visit record. Additionally, we created a new feature called “EHR length” which measured and recorded the difference between these two dates in days and encompassing EHR data from 287,012 participants in the workbench.^27^

Next, we queried the demographic data for 413,457 participants and extracted date of birth, age, sex assigned at birth, race, and ethnicity of participants. Subsequently, demographic information was added to the case and control datasets. Following this addition, these datasets were merged, and a new feature called “age at event” was created. For participants in the case cohort, this feature indicated their age at dementia onset. For controls, this reflected their age at the most recent EHR registration. The merged data at this stage contained another column called “dementia status.” This is the column that indicated whether a participant belonged to the cases cohort or the controls.

**RESEARCH‐IN‐CONTEXT**

1. Systematic review: We surveyed the literature through well‐established sources such as PubMed. It has been suggested that Hispanic/Latino and non‐Hispanic Black individuals have a higher risk of dementia and an attenuated impact of the apolipoprotein ε4 allele (APOE4) on their Alzheimer's disease (AD) risk. We sought to characterize the risk of all‐cause dementia and impact of APOE4 on the risk of developing a diagnosis of dementia in numerous participants from three ethnoracial groups in the *All of Us* Research Program.
2. Interpretation: Compared to non‐Hispanic White individuals, Hispanic/Latino and non‐Hispanic Black individuals are at higher risk of developing a “dementia diagnosis” and comparably higher risk in APOE4 carriers than in non‐carriers.
3. Future directions: Plasma biomarkers could inform the risk of AD versus non‐AD dementias and impact of APOE4 on AD in these ethnoracial groups.

Lastly, since our study aimed to investigate late onset AD specifically, we tried to get as close as possible to true cases by further filtering for participants with “age at event” that occurred at ages 60 or older to try and remove possible early‐onset cases. After final filtering, 85,109 individuals met criteria, and the final cohort comprised 2,459 diagnosed cases and 82,650 controls (Table 1, Figure S1).

**TABLE 1:** Total number, proportion, and ages of APOE4 allele copy groups in the overall cohort stratified by ethnorace, sex, and total number of all‐type dementia cases and controls.

		APOE4 allelic copy			Cases and controls	
Parmater		Non‐carrier (2/2 + 2/3 + 3/3)	Heterozygote (2/4 + 3/4)	Homozygote (4/4)	Cases^a^	Controls
**Overall, *N* (%)**	85,109	62,431 (73.4%)	20,884 (24.5%)	1,794 (2.1%)	2,459 (2.9%)	82,650 (97.1%)
Age at event, M (± SD)	70.6 (± 7.4)	70.8 (± 7.5)	70.0 (± 7.1)	68.7 (± 6.5)	72.8 (± 8.0)	70.5 (± 7.4)
Current age, M (± SD)	71.7 (± 7.5)	71.9 (± 7.5)	71.1 (± 7.2)	69.9 (± 6.7)	77.8 (± 7.8)	71.5 (± 7.4)
**Ethnoracial group, *N* (%)**						
Non‐Hispanic Black	14,937	9,324 (62.4%)	4,986 (33.4%)	627 (4.2%)	317 (2.1%)	14,620 (97.9%)
Hispanic/Latino	9,784	7,585 (77.5%)	2,063 (21.1%)	136 (1.4%)	406 (4.1%)	9,378 (95.9%)
Non‐Hispanic White	60,388	45,522 (75.4%)	13,835 (22.9%)	1,031 (1.7%)	1,736 (2.9%)	58,652 (97.1%)
**Sex, *N* (%)** ^b^						
Female	47,555	34,929 (73.4%)	11,673 (24.5%)	953 (2.0%)	1,322 (2.8%)	46,233 (97.2%)
Male	36,488	26,717 (73.2%)	8,957 (24.5%)	814 (2.2%)	1,100 (3.0%)	35,388 (97.0%)

*Note*: Counts with percentages and man (M) and standard deviation (± SD) for age at event and current age in APOE4 non‐carriers (all with 2/2, 2/3, and 3/3 genotypes), APOE4 heterozygotes (all with 2/4 and 3/4 genotype), and APOE4 homozygotes for the overall cohort, are reported.

Abbreviations: AD, Alzheimer's disease; APOE4, apolipoprotein *ε4*.

^a^ Cases included those with a diagnosed of any type of dementia (e.g., uncomplicated senile dementia, AD, etc.) or were prescribed cholinesterase inhibitors and/or glutamate regulators such as donepezil, rivastigmine galantamine and memantine.

^b^ There were 1066 missing values for sex at birth variable that were either skipped or participants preferred not to answer.

### 2.4 Statistical analyses

We characterized and compared the differential risk of progressing to a clinical diagnosis of all‐cause dementia in APOE4 carriers, including APOE4 homozygotes and heterozygotes (all with the APOE 2/4 and 3/4), and non‐carriers (all with the APOE 2/2, 2/3, and 3/3 genotype) in NHB, Hispanic/Latino, and NHW *All of Us* research participants. We used Cox proportional hazards ratio (HR) and 95% confidence intervals (CIs)^28^, ^29^ with data from participants with each of these genotypes and chi‐squared to compare APOE4 allelic frequency in the ethnoracial and sex groups. The “age at event” feature, as described above, was used as the dependent time variable in the survival analyses for participants with the event at ages 60 and older. For exploratory analyses and to address potential confounding factors, we tested the effect of social determinants of health (SDoH) separately and in combination with APOE4 allele copy and ethnoracial group using a deprivation index constructed from socioeconomic data using six American Community Survey (ACS) measures linked to the three‐digit zip code of participants and included variables such as fraction of assisted income and fraction of high school education for the weighted population average.^30^ We binned the continuous deprivation index variable into tertiles [0–33% low (1), 34%–66% medium (2), 67%–and above high (3)]. Similarly, we explored the potential associations with those who had sex at birth information reported. We did not include in this analysis participants who either skipped, chose not to disclose, or selected something other than male or female (intersex). First, we examined the effects APOE4 allelic dose in the three separate ethnoracial groups (NHB, Hispanic/Latino, NHW), and explored other variables of interest such as deprivation index and sex. As post hoc, we further examined these variables of interest including ethnorace, in the overall cohort. Additionally, we evaluated the significance of some interesting interaction terms (e.g., between APOE4 allelic copy and ethnoracial group) in the overall cohort using the likelihood ratio test.

## 3 RESULTS

### 3.1 Demographic characteristics

Demographic characteristics of the 85,109 individuals that met criteria described above and formed the study population, including age of first diagnosis of dementia or when first started on the dementia‐related medication (i.e., age at event), current age of participants, total number and proportion of APOE4 gene dose groups further stratified by ethnorace, sex, and total number of all‐type dementia cases and controls, are shown in Table 1. We used the self‐reported race and ethnicity information and had 9,784 Hispanic/Latino, 14,937 NHB, and 60,388 NHW individuals in each ethnoracial group. Chi‐squared test comparing the APOE4 allele frequency in the ethnoracial groups was significantly different (χ^2^ = 1268.5, df = 4, *p* < 2.2e^−16^), with NHB individuals having the highest proportion of APOE4 homozygotes (4.2%)—more than twice that of NHW individuals (1.7%) and nearly three times that of Hispanic/Latino individuals (1.4%). Additionally, NHB individuals had the highest proportion of APOE4 heterozygotes (33.4%), while Hispanic/Latino individuals had the highest proportion of APOE4 non‐carriers (77.5%), suggesting a lower APOE4 prevalence in this group (Table 1). Comparison for APOE4 allele frequency and sex was not significant. Ages and percentages of all‐cause dementia cases and controls in NHB, Hispanic/Latino, and NHW APOE4 homozygotes, heterozygotes (all with APOE 2/4 or 3/4 genotype), and APOE4 non‐carriers (all with APOE 2/2, 2/3, or 3/3 genotype) ages 60*+ All of Us* participants are shown in Table 2.

**TABLE 2:** Ages and percentages of all‐cause dementia cases and controls stratified by APOE4 allele copies and ethnoracial group.

	Ethnoracial groups			ANOVA/chi‐squared		
	Non‐Hispanic Black	Hispanic/Latino	Non‐Hispanic White	NHW vs. NHB	NHW vs. H/L	H/L vs. NHB
Parameter	(*n* = 14,937)	(*n* = 9,784)	(*n* = 60,388)	*p*‐value	*p*‐value	*p*‐value
**Overall**						
Age at event, M (± SD)	67.3 ± 6.1	68.4 ± 6.6	71.7 ± 7.5	<0.0001	<0.0001	<0.0001
Current age, M (± SD)	68.3 ± 6.2	69.6 ± 6.8	72.8 ± 7.5	<0.0001	<0.0001	<0.0001
**Cases** **(%)** ^a^						
Age at event, M (± SD)	69.9 (± 7.4)	72.6 (± 7.9)	73.4 (± 8.1)	1.09e^−13^	0.20	8.28e^−6^
Age current, M (± SD)	74.9 (± 7.6)	78.0 (± 7.5)	78.3 (± 7.8)	1.28e^−12^	1.00	1.83e^−7^
APOE4 homozygotes	5.0%	2.0%	4.2%	4.56e^−9^	1.53e^−12^	0.30
APOE4 heterozygotes	36.0%	27.8%	26.6%	3.63e^−47^	1.64e^−47^	1.00
APOE4 non‐carriers	59.0%	70.2%	69.2%	1.21e^−162^	2.46e^−124^	1.93e^−5^
**Controls (%)**						
Age at event, M (± SD)	67.2 (± 6.0)	68.2 (± 6.5)	71.7 (± 7.4)	<0.0001	<0.0001	<0.0001
Age current, M (± SD)	68.2 (± 6.1)	69.2 (± 6.5)	72.7 (± 7.5)	<0.0001	<0.0001	<0.0001
APOE4 homozygotes	4.2%	1.4%	1.6%	5.84e^−18^	1.70e^−139^	3.78e^−70^
APOE4 heterozygotes	33.3%	20.8%	22.8%	<0.0001	<0.0001	1.14e^−273^
APOE4 non‐carriers	62.5%	77.8%	75.6%	<0.0001	<0.0001	4.36e^−46^

*Note*: Cases and controls in non‐Hispanic Black (NHB), Hispanic/Latino, and non‐Hispanic White (NHW) APOE4 homozygotes, heterozygotes (all with APOE 2/4 or 3/4 genotype), and non‐carriers (all with APOE 2/2, 2/3, or 3/3 genotype) ages 60*+ All of Us* participants. Mean (M) and standard deviation (± SD) for age at event and current age with corresponding Bonferroni adjusted ANOVA pairwise comparisons are reported. Chi‐squared tests for the proportion of cases (χ^2^ = 18.1, df = 4, *p* = 0.001) and for the proportion of controls (χ^2^ = 1280.5, df = 4, *p* < 2.2e^−16^) were significant, pairwise chi‐squared with Bonferroni adjusted *p*‐values for the proportions of APOE4 homozygotes, heterozygotes, and non‐carriers in cases and controls across the three ethnoracial groups are reported.

Abbreviations: AD, Alzheimer's disease; ANOVA, analysis of variance; APOE4, apolipoprotein *ε4*.

^a^ Cases included those with a diagnosed of any type of dementia (e.g., uncomplicated senile dementia, AD, etc.) or were prescribed cholinesterase inhibitors and/or glutamate regulators such as donepezil, rivastigmine galantamine and memantine.

### 3.2 Analyses in the individual ethnoracial groups

Results showed that APOE4 increased dementia diagnosis risk significantly in all groups and by allelic copy, but effects varied by race and ethnicity.

**FIGURE 1:**
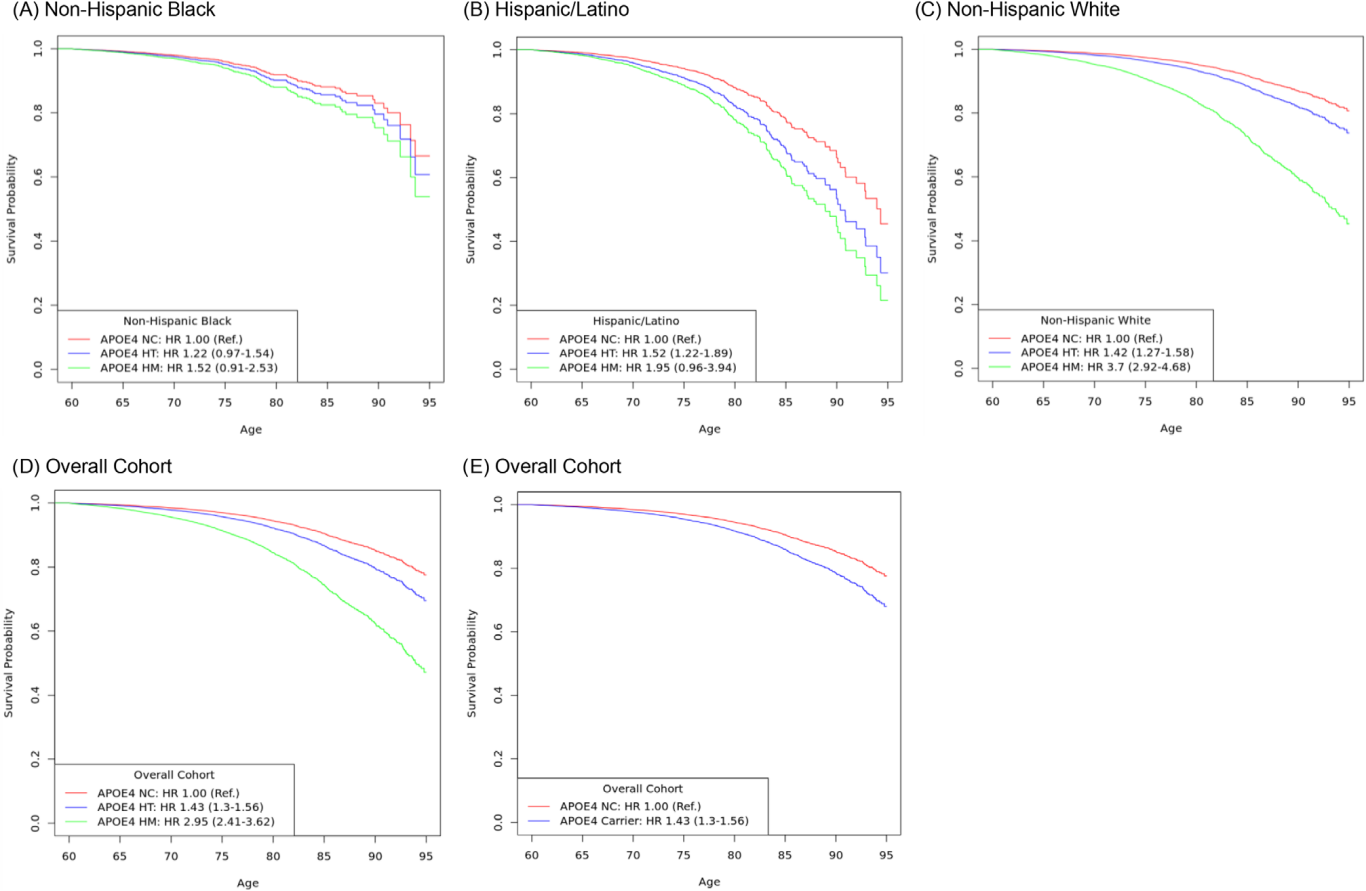
Survival curves for APOE4 allele copy in *All of Us p*articipants across ethnoracial groups. Kaplan‐Meier survival curves for APOE4 non‐carriers (APOE4 NC, red), heterozygotes (APOE4 HT, Blue), and homozygotes (APOE4 HM, green) in *All of Us* participants from three ethnoracial groups: (A) non‐Hispanic Black, (B) Hispanic/Latino, and (C) non‐Hispanic White. Additionally, survival curves are shown for (D) the overall cohort and (E) all APOE4 carriers combined (blue). APOE4, apolipoprotein *ε4*; HM, homozygote; HT, heterozygote.

Analysis in the 14,937 NHB individuals showed that the combined APOE4 carrier group had a significant increased risk for having a dementia diagnosis while the individual NHB APOE4 heterozygote and homozygote groups showed a non‐significant trend toward increased risk compared to the NHB non‐carrier reference group (APOE4 carriers: HR 1.25, 95% CI 1.00–1.57, *p* = 0.04; heterozygote: HR 1.22, 95% CI 0.97–1.54, *p* = 0.09; homozygote: HR 1.52, 95% CI 0.91–2.53, *p* = 0.10, Figure 1A and Table S1).

In the 9,784 Hispanic/Latino individuals, combined APOE4 carriers and Hispanic/Latino heterozygote groups also exhibited significant risk versus the Hispanic/Latino non‐carrier reference group, however, in the Hispanic/Latino homozygote group, differences only trended toward significance (APOE4 carriers: HR 1.54, 95% CI 1.25–1.91, *p* = 6.5e^−5^; heterozygote: HR 1.52, 95% CI 1.22–1.89, *p* = 1.7e^−4^; homozygote: HR 1.95, 95% CI 0.96–3.94, *p* = 0.06, Figure 1B and Table S1).

In the 60,388 NHW individuals, risk increased significantly with APOE4 allelic copy number and for the combined NHW APOE4 carrier group compared with the NHW non‐carrier reference group (heterozygote: HR 1.42, 95% CI 1.27–1.58, *p* = 1.6e^−10^; homozygote: HR 3.70, 95% CI 2.92–4.68, *p* = < 2e^−16^; APOE4 carriers: HR 1.55, 95% CI 1.40–1.72, *p* = < 2e^−16^, Figure 1C and Table S1).

We found consistent results when dementia‐related prescriptions without a corresponding dementia diagnostic code and postconcussion syndrome cases were removed from the phenotyping algorithm, further strengthening the findings, particularly for the APOE4 homozygote groups (Table S2).

### 3.3 APOE4 and ethnoracial group analyses in the overall cohort

In the overall cohort with the 85,109 participants, risk increased significantly with APOE4 allelic dose (heterozygote: HR 1.43, 95% CI 1.30–1.56, *p* = 5.4e^−15^; homozygote: HR 2.95, 95% CI 2.41–3.63, *p* = < 2e^−16^ versus non‐carriers, Figure 1D) and by APOE4 carriership (APOE4 carriers: HR 1.52, 95% CI 1.40–1.66, *p* = < 2e^−16^; versus APOE4 non‐carrier reference group, Figure 1E).

**FIGURE 2:**
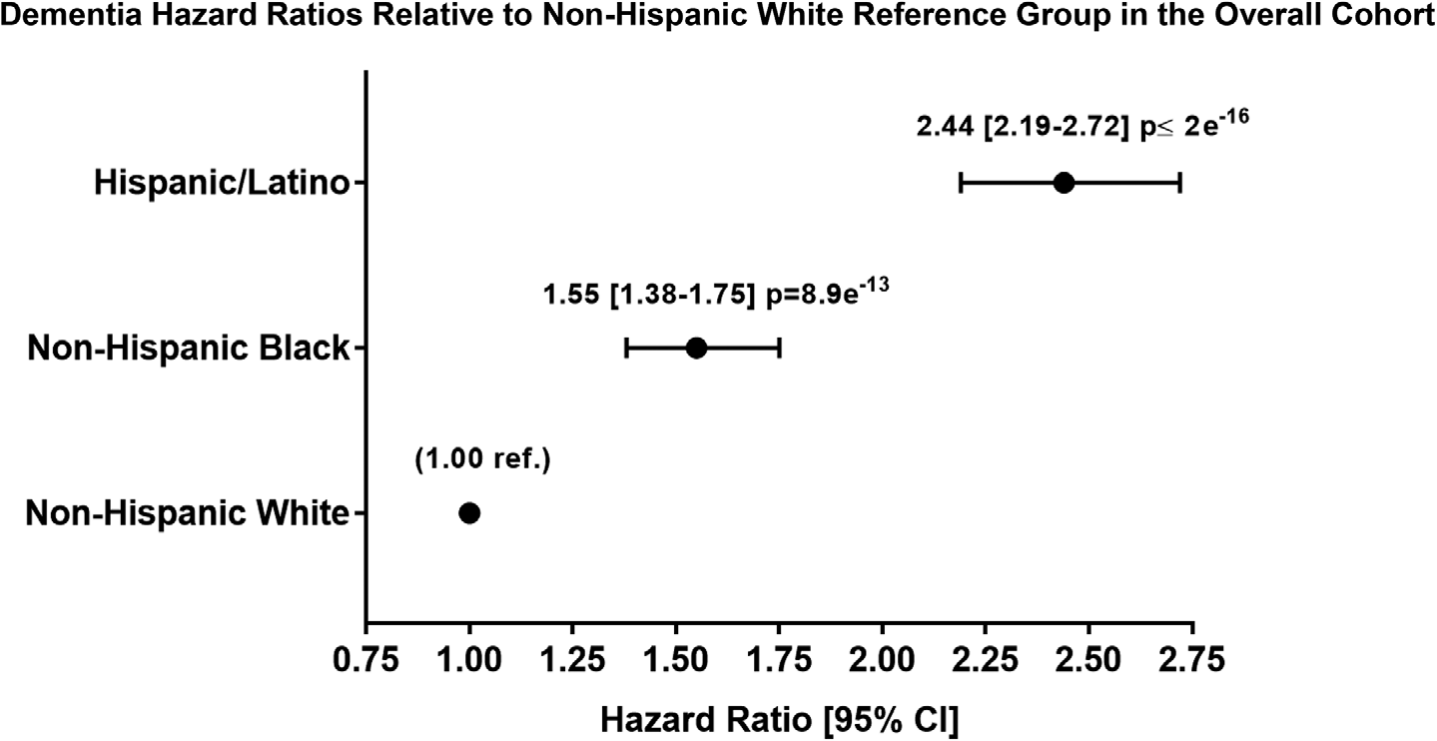
Dementia hazard ratios relative to non‐Hispanic White reference group in the overall cohort. Cox proportional hazard ratio with 95% confidence intervals (CIs) in Hispanic/Latino and non‐Hispanic Black compared with the non‐Hispanic White reference group (1.00 ref.) from the overall cohort.

Independent from APOE4 status, results indicate that the Hispanic/Latino group had a dementia diagnosis risk that was 144% higher followed by the NHB group with a risk that was 55% higher compared with the NHW reference group (Hispanic/Latino: HR 2.44, 95% CI 2.19–2.72, *p* = < 2e^−16^; NHB: HR 1.55, 95% CI 1.38–1.75, *p* = 8.9e^−13^, Figure 2). Given that the individual results suggest the simultaneous effect of APOE4 and ethnorace is important in survival, we tested the significance of the interaction effect between these two variables using a likelihood ratio test for a comparison between a full model with an interaction term and a reduced model without interaction. The test statistic showed the interaction term was significant (χ^2^ = 13.60, df = 4, *p* = 0.008). We again found stronger, more consistent results with the restricted case definition for the sensitivity analyses mainly in the APOE4 homozygote group (Table S2). The likelihood ratio test for this interaction was no longer significant (*p* = 0.06).

### 3.4 Deprivation index analyses

**FIGURE 3:**
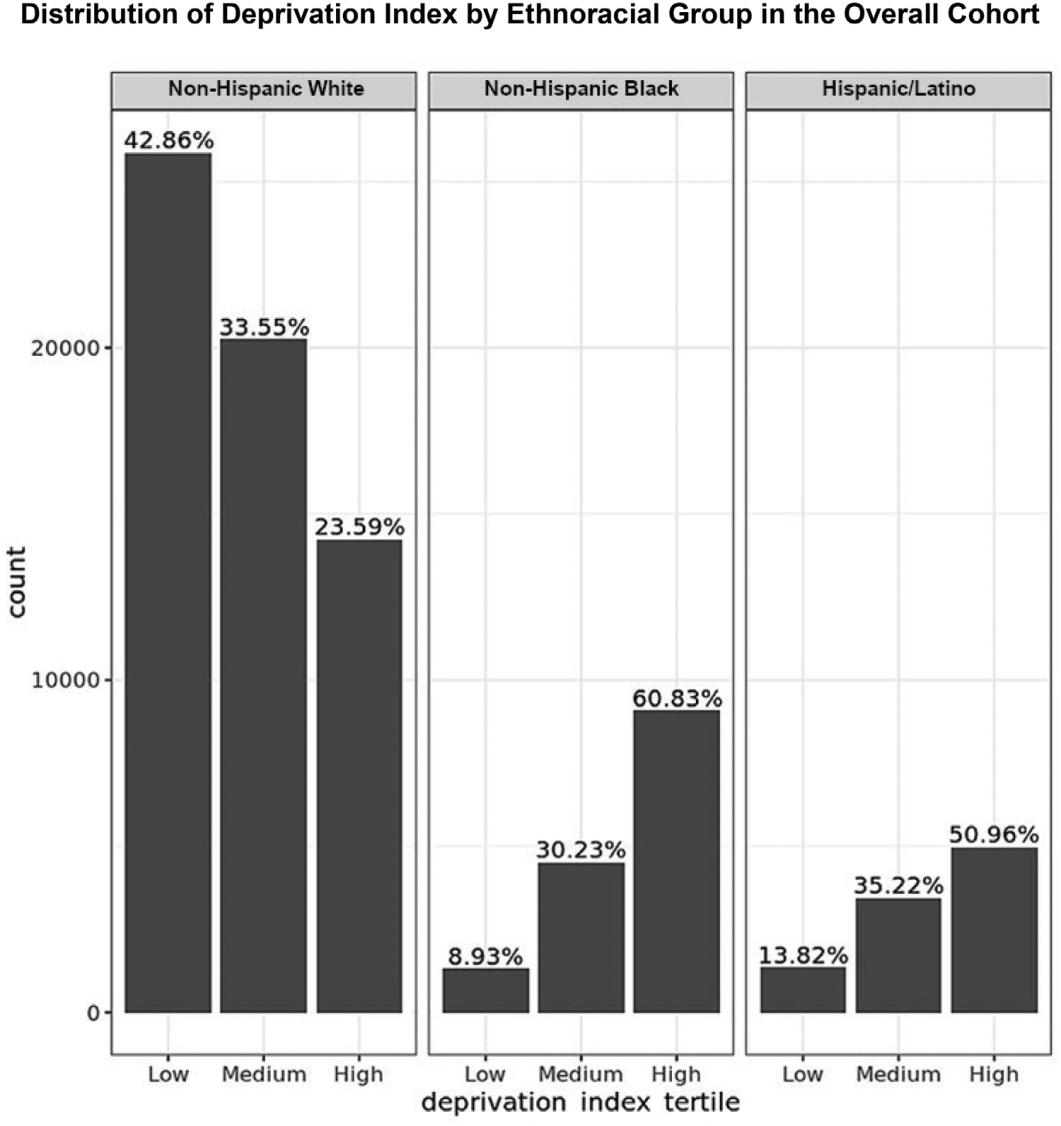
Distribution of deprivation index by ethnoracial group in the overall cohort. Percentages for low, medium, and high deprivation index tertiles in non‐Hispanic White, non‐Hispanic Black, and Hispanic/Latino groups from the overall cohort.

Distribution of low, medium, and high deprivation index tertiles across the three ethnoracial groups is shown in Figure 3. Briefly, low deprivation was highest in the NHW population (∼ 43%) while high deprivation accounted for ∼ 61% of the NHB population and ∼ 51% of the Hispanic/Latino population in this study (Figure 3). Cox proportional HR of deprivation index tertiles in the individual ethnoracial groups was significant for medium deprivation relative to the low deprivation reference group (Table S1). Analysis of deprivation index in the overall cohort indicated a 48% increased risk of receiving a dementia diagnosis for the medium deprivation tertile and 27% increased risk for the high deprivation tertile when compared with the low deprivation reference group (medium deprivation index tertile: HR 1.48, 95% CI 1.34–1.62, *p* = 5.7e^−16^; high deprivation index tertile: HR 1.27, 95% CI 1.15–1.41, *p* = 4.1e^−6^). Furthermore, we investigated the significance of the interaction between deprivation index and APOE4 allele copy and the interaction between deprivation index and ethnoracial groups. The likelihood ratio test for the interaction with APOE4 allele copy was not significant (*p* = 0.55), but they were significant for the interaction with ethnoracial group (χ^2^ = 13.80, df = 4, *p* = 0.007). Results for the sensitivity analysis are detailed in the Supplement.

### 3.5 Sex analyses

Analysis of sex in the individual ethnoracial groups was not significant as is shown in Table S1. Analysis in the 47,555 women and 36,488 men in the overall cohort with sex as a single covariate was not significant (sex: HR 0.94, 95% CI 0.87–1.02, *p* = 0.13). In a complementary test, APOE4 allele copy was added as an additional covariate to the model. Consistent with the previous outcomes and as expected, the effect of sex was still insignificant (sex: HR 0.94, 95% CI 0.87–1.02, *p* = 0.11), while the main effect of APOE4 allele copy remained significant (heterozygote: HR 1.44, 95% CI 1.32–1.57, *p* = 1.4e^−15^; homozygote: HR 2.95, 95% CI 2.40–3.62, *p* = < 2e^−16^ compared with APOE4 non‐carrier reference group). The likelihood ratio test for the interaction with APOE4 allele copy was not significant (*p* = 0.08).

## 4 DISCUSSION

Using diagnostic codes in over 85,000 *All of Us* participants, we observed a greater risk of getting a dementia diagnosis for the Hispanic/Latino and NHB populations compared with the NHW population in this study. We also observed a comparably elevated risk of APOE4 in Hispanic/Latino and NHB groups compared to the NHW population.

Furthermore, we found that those in the medium deprivation tertile, which are disproportionally from URGs, are at higher risk of a dementia diagnosis compared to those in the low deprivation index tertile. Interestingly, the highest deprivation tertile did not show the same increased risk, which may reflect a non‐linear relationship between deprivation and dementia. This could be due to factors such as lower healthcare access and underdiagnosis in extremely deprived populations, rather than a true lower risk.^31^, ^32^ Additionally, survivor bias may contribute, as individuals with the greatest socioeconomic disadvantages often face higher mortality before a dementia diagnosis can be made.^33^ Other potential explanations include cognitive reserve effects, where variations in educational and occupational exposure influence dementia risk differently across deprivation levels,^34^, ^35^ and disparities in healthcare access leading to underdiagnosis in the most deprived populations. These findings indicate that factors beyond genetics, such as SDoH, are significant predictors of dementia risk and should be further examined to better understand associations with APOE4 and modifiable risk factors, ultimately supporting the development of future prevention therapies.

Among different racial and ethnic groups, studies have shown the impact of APOE4 varies, with African American and Hispanic/Latino individuals typically showing lower risk of AD dementia compared to non‐Hispanic White individuals.^14^, ^15^, ^16^, ^17^, ^18^, ^19^, ^20^ In our study, the stronger APOE4 dosage effect observed in Hispanic/Latino individuals compared to non‐Hispanic Black individuals may reflect differences in ancestry admixture, including varying proportions of Amerindian, African, and European genetic backgrounds, which have been shown to influence APOE4‐associated AD risk.^19^ Additionally, as we used self‐reported race and ethnicity rather than genetic ancestry, our findings may be influenced by heterogeneity within the Hispanic/Latino population, where ancestry composition can differ significantly across cohorts and impact risk estimates.

Although we did not see any effects of sex, women with APOE4 tend to have higher incident dementia risk hazard compared to men.^36^ While this varies, most studies suggest that APOE4 effects on AD risk are greater in women and some suggest an age‐by‐sex specific interaction where APOE4 heterozygote women have an increased risk at younger ages.^14^, ^19^, ^36^, ^37^, ^38^, ^39^

Limitations in detecting stronger APOE4 allelic dose effects or interactions with these URGs could also be related to differences in sample size of APOE4 carriers in the URGs, accuracy to characterize AD cases and controls in EHR datasets, underestimating AD HRs and interactions due to misdiagnosed cases, inclusion of controls who may have preclinical AD, potential biases in cognitive and clinical assessments, biases in detecting impairment in the different ethnoracial groups and actual differences in the percent of impaired with AD versus non‐AD pathology (e.g., cerebrovascular disease). Other possible reasons for these lower‐than‐expected HRs with regard to other studies could be due to the younger age skew and limited number of AD cases in this cohort. We highlight the importance of the accuracy of diagnoses matter when it comes to the magnitude of these effects by noting that the HRs are much lower than expected for AD.^6^, ^19^, ^27^

For this study, we looked at the prevalence of all‐cause dementia, the impact of APOE4 allele copy in these URGs, and sought to compare them. We found that these URGs have a higher rate of dementia diagnosis compared to the NHW population^40^ while we could not show with significance that the effects of APOE4 were attenuated in URGs for the following reasons. First, this study highlights the limitations of using an EHR cohort for analyzing all‐cause dementia, particularly due to the difficulty in differentiating AD from other causes of dementia and the differential effect of APOE4 allelic dose across ethnoracial groups. Additionally, site‐specific patterns in billing practices, data uploads, and potential differences in cognitive and clinical assessments, might have introduced confounding factors. We note that our data analysis initially excluded APOE2 carriers (2/2, 2/3, and 2/4) but later included them to increase sample sizes across groups of interest, acknowledging that this approach does not account for the protective effects of APOE2.^4^, ^6^, ^9^ We also note significant differences in the observation period with NHWs having longer time‐at‐risk for the event but clarify that this was accounted for in the survival analyses by using time‐to‐event as the outcome (versus Yes/No for dementia status) and running the analyses in the individual ethnoracial groups. Similarly, while we included several major SDoH, our investigation was not comprehensive, as it was limited to factors assessed in the majority of *All of Us* participants. Future inclusion of more detailed SDoH data could help disentangle biological from sociocultural influences on disease risk. Based on these limitations, we propose using plasma pTau217 as an endophenotype of AD^41^, ^42^ within *All of Us* to better characterize AD prevalence, assess the differential risk of APOE4 allelic dose, and clarify the proportions of AD versus non‐AD dementias in each group. Finally, we acknowledge the potential value of evaluating the extremely rare APOE ε1 allele and emphasize the importance of refining clinical diagnoses, which can be biased, by incorporating biomarkers to address these limitations and clarify these critical issues. We plan to characterize and compare the impact of each APOE genotype, and the number of APOE4 and APOE2 alleles in a person's genotype, on the incidence versus prevalence of dementia in several hundred thousand *All of Us* participants.

There is a growing rate of dementia especially in Hispanic/Latino and NHB populations, but despite that, there is evidence showing a potentially lower impact of APOE4 in these URGs compared to the NHW population.^14^, ^15^, ^16^, ^17^, ^18^, ^19^, ^20^ We capitalized on an unusually large cohort enriched for URGs to look at the prevalence of all‐cause dementia. Clarifying any differences in the effects of the APOE4 allele among these different populations (i.e., support findings from smaller case control studies), would (1) help to inform persons from these URGs about their risk of developing AD and possibly increase their participation in clinical studies^43^, (2) support efforts to clarify the potentially targetable protective factors that account for their differential risk, and (3) set the stage to determine whether recently established disease monoclonal treatments have comparable benefits and risks in mildly impaired persons with biomarker evidence of AD from Hispanic/Latino and Black/African American backgrounds.

## CONFLICT OF INTEREST STATEMENT

V.G., E.K., I.S.P., M.H.M‐A., H.D.P., D.D.G., Y.C., M.N., D.S., O.J.V., C.O.M., Y.S., M.J.H., and J.H.K., report no disclosures. E.M.R is a principal investigator of the NIH‐supported University of Arizona‐Banner Health *All of Us* Research Program, other studies that are supported by NIH, state of Arizona, and philanthropic grants, and prevention trials that receive NIH, industry (Lilly and Roche), and/or philanthropic support. He is a co‐founder, advisor and shareholder in ALZpath, a company that involved in the development and use of Alzheimer's disease biomarkers, including plasma pTau217, and an inventor of several patents that are not related to this study. He is a compensated advisor to Alzheon, Denali, Cognition Therapeutics, Enigma, Retromer Therapeutics, and Vaxxinity.

## CONSENT STATEMENT

The study was approved by the Institutional Review Board (IRB), and informed consent was obtained for all participants. All experimental protocols involving human participants were approved by the Ethics Committee and IRB of the *All of Us* Research Program. For more details see *All of Us* Policy on the Ethical Conduct of Research.

## Supporting information



Supporting Information

Supporting Information

## Data Availability

This study used data from the *All of Us* Research Program's Controlled Tier Dataset version number 7, available to authorized users on the Researcher Workbench. The *All of Us* data use agreement strictly prohibits researchers from sharing row‐level data. Access to data from *All of Us* is limited to the *All of Us* Research Workbench, as outlined in the participants' informed consent. All data and analysis source code for this project are hosted within the *All of Us* Workbench and will be accessible to any approved *All of Us* researcher. https://workbench.researchallofus.org/workspaces/aou‐rw‐ee111e87/impactofapoe4onsurvivalfromdiagnosisofaddementiaindiversepopulations.
