# Supplementary material for: Impact of APOE4‐related dementia risk in underrepresented groups from the All of Us research program
Source: Alzheimers Dement. 2025 Jun 11;21(6):e70245. doi: 10.1002/alz.70245 (PMC12159303; doi:10.1002/alz.70245)
Supplement: Supplementary file 1 — Supporting Information [file ALZ-21-e70245-s001.docx]

**Impact of APOE4-related dementia risk in underrepresented groups from the *All of Us* research program**

**Authors:**

Valentina Ghisays^1^*, Ehsan Khajouei^2*^, Ignazio S. Piras^3^, Michael H. Malek-Ahmadi^1^, Hillary D. Protas^1^, Dhruman D. Goradia^1^, Yinghua Chen^1^, Marcus Naymik^3^, Donald Saner^1^, Olivia J. Veatch^4^, Clayton O. Mansel^4^, Yi Su^1^, Matthew J. Huentelman^3^, Jason H. Karnes ^2^*, Eric M. Reiman^1,3*^

*V.G & E.K are co-first authors; J.H.K & E.M.R are co-senior authors

**Affiliations:**

^1^ Banner Alzheimer’s Institute, Phoenix, AZ, USA

^2^ Department of Pharmacy Practice and Science, R. Ken Coit College of Pharmacy, University of Arizona, Tucson, AZ, USA

^3^ Early Detection and Prevention Division, Translational Genomics Research Institute, Phoenix, AZ, USA

^4^ Department of Cell Biology and Physiology, University of Kansas Medical Center, 3901 Rainbow Blvd, Kansas City, KS, 66160

# **SUPPLEMENTARY RESULTS**

## Deprivation Index Analyses

We found similar results, with a few differences, when dementia-related prescriptions without a corresponding dementia diagnostic code and Postconcussion syndrome cases were removed from the phenotyping algorithm. Cox proportional HR of deprivation index tertiles in the individual ethnoracial groups was again significant for medium deprivation relative to the low deprivation reference group except in the individual Non-Hispanic Black (NHW) group (p = 0.13, **Supplementary Table 2**). The other difference in the individual ethnoracial group analyses was found in the Non-Hispanic White (NHW) group with now significant increased risk for the high deprivation index compared to the low deprivation reference group (p = 0.02, **Supplementary Table 2**).

Analysis of deprivation index in the overall cohort indicated a 54% (vs. 48%) increased risk of receiving a dementia diagnosis for the medium deprivation tertile and a 48% (vs. 27%) increased risk for the high deprivation tertile when compared with the low deprivation reference group (**Supplementary Table 2**). We investigated the significance of the interaction between deprivation index and APOE4 allele copy, and it remained not significant (p = 0.5). The likelihood ratio test for the interaction with deprivation index and ethnoracial group was now no longer significant (p = 0.06).

**Supplementary Figure 1. Data flow chart for building the cohort on the Workbench**

**
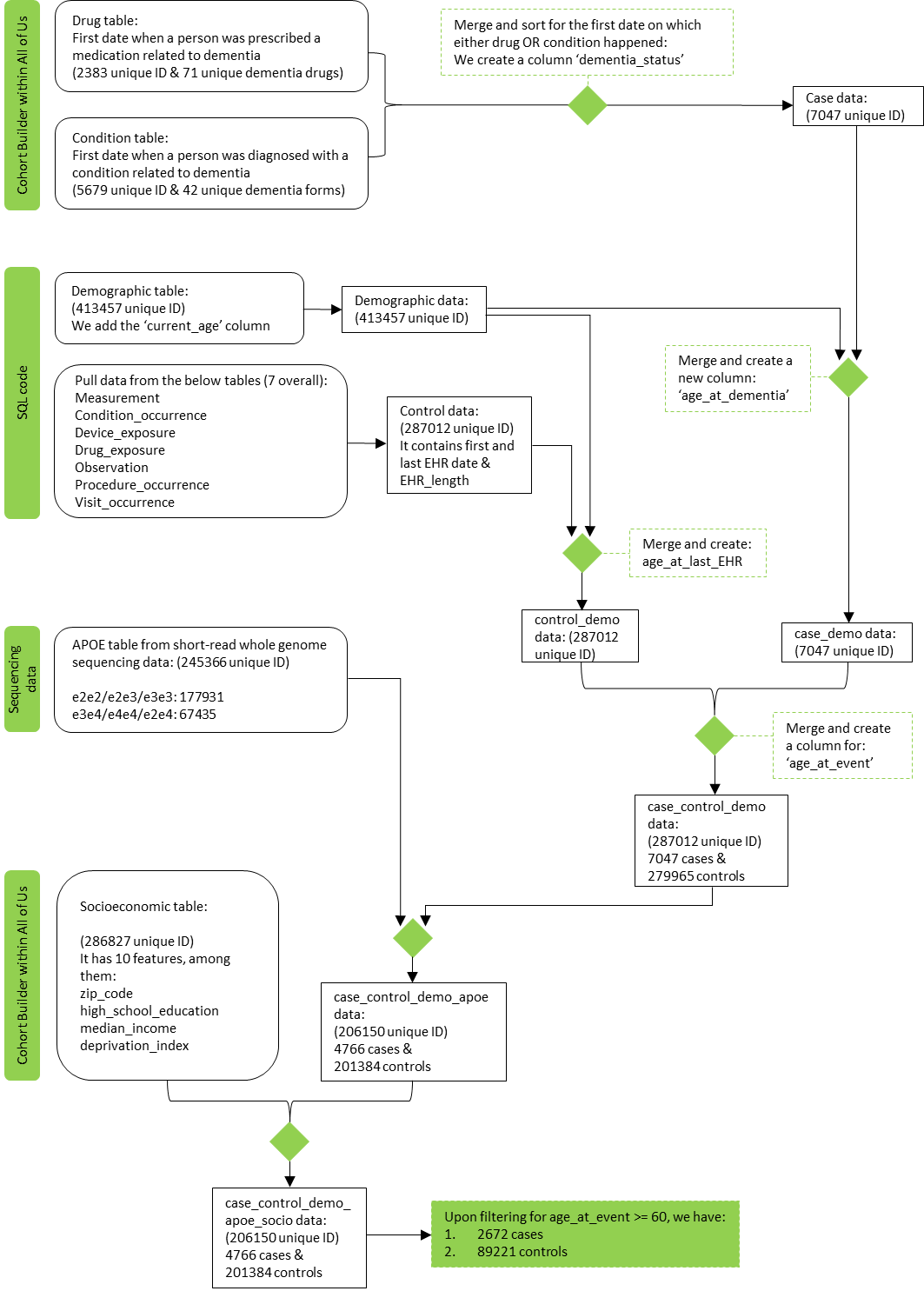
**

**Supplementary Table 1.** Survival Analyses Results Across APOE4 Allele Copy and Carriership Further Stratified by Deprivation Index and Sex in Non-Hispanic Black, Hispanic/Latino and Non-Hispanic White *All of Us* Participants.

|  | **Non-Hispanic Black** | | | | |  | | | **Hispanic/Latino** | | | | | | | **Non-Hispanic White** | | | | | | | **Overall Cohort** | | | | | |
| --- | --- | --- | --- | --- | --- | --- | --- | --- | --- | --- | --- | --- | --- | --- | --- | --- | --- | --- | --- | --- | --- | --- | --- | --- | --- | --- | --- | --- |
| **Model** | (n) |  | HR (95% CI) |  | P-value | |  | (n) | |  | HR (95% CI) |  | P-value |  | (n) | |  | HR (95% CI) |  | P-value |  | (n) | |  | HR (95% CI) |  | P-value |  |
| APOE4 Carrier  (2/4 + 3/4 + 4/4) | 5,613 |  | 1.25 (1.00-1.57) |  | 0.04 | |  | 2,199 | |  | 1.54 (1.25-1.91) |  | 6.5e^-5^ |  | 14,866 | |  | 1.55 (1.40-1.72) |  | < 2e^-16^ |  | 22,678 | |  | 1.52 (1.40-1.66) |  | < 2e^-16^ |  |
| Non-Carrier  (2/2 + 2/3 + 3/3) | 9,324 |  | Ref. |  | - | |  | 7,585 | |  | Ref. |  | - |  | 45,522 | |  | Ref. |  | - |  | 62,431 | |  | Ref. |  | - |  |
| Heterozygotes  (2/4 + 3/4) | 4,986 |  | 1.22 (0.97-1.54) |  | 0.09 | |  | 2,063 | |  | 1.52 (1.22-1.89) |  | 1.7e^-4^ |  | 13,835 | |  | 1.42 (1.28-1.58) |  | 1.6e^-10^ |  | 20,884 | |  | 1.43 (1.30-1.56) |  | 5.4e^-15^ |  |
| Homozygotes  (4/4) | 627 |  | 1.52 (0.91-2.53) |  | 0.10 | |  | 136 | |  | 1.95 (0.96-3.94) |  | 0.06 |  | 1,031 | |  | 3.70 (2.92-4.68) |  | < 2e^-16^ |  | 1,794 | |  | 2.95 (2.41-3.63) |  | < 2e^-16^ |  |
| **Deprivation Index**  **Tertiles ^a^** |  |  |  |  |  | |  |  | |  |  |  |  |  |  | |  |  |  |  |  |  | |  |  |  |  |  |
| Medium | 4,511 |  | 1.49 (1.00-2.20) |  | 0.04 | |  | 3,440 | |  | 1.80 (1.26-2.57) |  | 0.001 |  | 20,254 | |  | 1.25 (1.12-1.39) |  | 5.2e^-5^ |  | 28,205 | |  | 1.48 (1.34-1.62) |  | 5.7e^-16^ |  |
| Low | 1,333 |  | Ref. |  | - | |  | 1,350 | |  | Ref. |  | - |  | 25,871 | |  | Ref. |  | - |  | 28,554 | |  | Ref. |  | - |  |
| High | 9,077 |  | 0.86 (0.58-1.27) |  | 0.45 | |  | 4,977 | |  | 1.22 (0.85-1.75) |  | 0.27 |  | 14,239 | |  | 1.09 (0.96-1.24) |  | 0.16 |  | 28,293 | |  | 1.27 (1.15-1.41) |  | 4.1e^-6^ |  |
| **Sex ^b^** |  |  |  |  |  | |  |  | |  |  |  |  |  |  | |  |  |  |  |  |  | |  |  |  |  |  |
| Male | 14,607 |  | 1.04 (0.83-1.31) |  | 0.71 | |  | 9,677 | |  | 0.87 (0.71-1.07) |  | 0.18 |  | 59,759 | |  | 1.01 (0.91-1.11) |  | 0.90 |  | 36,488 | |  | 0.94 (0.87-1.02) |  | 0.13 |  |

Cox proportional hazards ratio (HR) with 95% CI and unadjusted p-values are reported. Significant p-values are highlighted in green.

^a^ There were 57 missing values for deprivation index tertiles:

- 16 observations deleted due to missingness in the Non-Hispanic Black
- 17 observations deleted due to missingness in the Hispanic/Latino
- 24 observations deleted due to missingness in the Non-Hispanic White

^b^ There were 1,066 missing values for sex:

- 330 observations deleted due to missingness in the Non-Hispanic Black
- 107 observations deleted due to missingness in the Hispanic/Latino
- 629 observations deleted due to missingness in the Non-Hispanic White

**Supplementary Table 2.** Sensitivity Survival Analyses Corresponding to Supplementary Table 1 Analyses in the Individual Ethnoracial Groups and in the Overall Cohort, Removing Cases of Dementia-Related Prescriptions Without a Corresponding Dementia Diagnostic Code and Postconcussion Syndrome Cases.

|  | **Non-Hispanic Black** | | | |  | | **Hispanic/Latino** | | | | | | **Non-Hispanic White** | | | | | | **Overall Cohort** | | | | |
| --- | --- | --- | --- | --- | --- | --- | --- | --- | --- | --- | --- | --- | --- | --- | --- | --- | --- | --- | --- | --- | --- | --- | --- |
| **Model** | (n) |  | HR (95% CI) |  | P-value |  | (n) |  | HR (95% CI) |  | P-value |  | (n) |  | HR (95% CI) |  | P-value |  | (n) |  | HR (95% CI) |  | P-value |
| APOE4 Carrier  (2/4 + 3/4 + 4/4) | 5,550 |  | 1.37 (1.02-1.84) |  | 0.03 |  | 2,158 |  | 1.75 (1.34-2.29) |  | 3.9e^-5^ |  | 14,626 |  | 1.78 (1.55-2.05) |  | 6.0e^-16^ |  | 22,334 |  | 1.74 (1.56-1.95) |  | < 2e^-16^ |
| Non-Carrier  (2/2 + 2/3 + 3/3) | 9,239 |  | Ref. |  | - |  | 7,467 |  | Ref. |  | - |  | 44,910 |  | Ref. |  | - |  | 61,616 |  | Ref. |  | - |
| Heterozygotes  (2/4 + 3/4) | 4,938 |  | 1.30 (0.96-1.78) |  | 0.09 |  | 2,022 |  | 1.66 (1.26-2.20) |  | 3.1e^-4^ |  | 13,621 |  | 1.59 (1.37-1.84) |  | 1.05e^-9^ |  | 20,581 |  | 1.59 (1.41-1.79) |  | 4.7e^-14^ |
| Homozygotes  (4/4) | 622 |  | 2.00 (1.07-3.72) |  | 0.02 |  | 136 |  | 3.28 (1.61-6.66) |  | 0.001 |  | 1,005 |  | 5.10 (3.78-6.85) |  | < 2e^-16^ |  | 1,763 |  | 4.10 (3.19-5.27) |  | < 2e^-16^ |
| **Deprivation Index**  **Tertiles ^a^** |  |  |  |  |  |  |  |  |  |  |  |  |  |  |  |  |  |  |  |  |  |  |  |
| Medium | 4,460 |  | 1.51 (0.88-2.59) |  | 0.13 |  | 3,365 |  | 1.71 (1.10-2.68) |  | 0.01 |  | 20,225 |  | 1.25 (1.07-1.45) |  | 0.004 |  | 28,050 |  | 1.54 (1.35-1.76) |  | 1.3e^-10^ |
| Low | 1,319 |  | Ref. |  | - |  | 1,337 |  | Ref. |  | - |  | 25,523 |  | Ref. |  | - |  | 28,179 |  | Ref. |  | - |
| High | 9,004 |  | 0.97 (0.57-1.66) |  | 0.91 |  | 4,907 |  | 1.16 (0.74-1.82) |  | 0.52 |  | 13,765 |  | 1.22 (1.03-1.45) |  | 0.02 |  | 27,676 |  | 1.48 (1.29-1.71) |  | 4.4e^-8^ |

Cox proportional hazards ratio (HR) with 95% CI and unadjusted p-values are reported. Significant p-values are highlighted in green.

^a^ There were 55 missing values for deprivation index tertiles

- 16 observations deleted due to missingness in the Non-Hispanic Black
- 16 observations deleted due to missingness in the Hispanic/Latino
- 23 observations deleted due to missingness in the Non-Hispanic White
